# Crop genome editing through tissue-culture-independent transformation methods

**DOI:** 10.3389/fgeed.2024.1490295

**Published:** 2024-12-05

**Authors:** Alejandro Sebiani-Calvo, Alejandro Hernández-Soto, Götz Hensel, Andrés Gatica-Arias

**Affiliations:** ^1^ Plant Biotechnology Laboratory, School of Biology, University of Costa Rica, San José, Costa Rica; ^2^ Programa de Posgrado en Biología, School of Biology, University of Costa Rica, San José, Costa Rica; ^3^ Biotechnology Research Center, Biology School, Costa Rica Institute of Technology, Cartago, Costa Rica; ^4^ Centre for Plant Genome Engineering, Institute for Plant Biochemistry, Heinrich-Heine University Düsseldorf, Düsseldorf, Germany; ^5^ Cluster of Excellence in Plant Sciences “SMART Plants for Tomorrow’s Needs”, Heinrich Heine University Düsseldorf, Düsseldorf, Germany

**Keywords:** agriculture, CRISPR, crop improvement, in-planta transformation, genome editing

## Abstract

Genome editing and plant transformation are crucial techniques in plant biotechnology, allowing for the precise modification of plant genomes to enhance agronomically essential traits. The advancement of CRISPR-based genome editing tools in plants is limited, among others, by developing novel *in vitro* tissue culture methodologies for efficient plant genetic transformation. *In-planta* methodologies offer a promising alternative to overcome tissue culture limitations and facilitate crops’ genetic improvement. The *in-planta* transformation methods can be categorized under the definition of means of plant genetic transformation with no or minimal tissue culture steps meeting the conditions for minimal steps: short duration with a limited number of transfers, high technical simplicity, limited list of hormones, and that the regeneration does not undergo callus development. In this review, we analyzed over 250 articles. We identified studies that follow an *in-planta* transformation methodology for delivering CRISPR/Cas9 components focusing on crop plants, as model species have been previously reviewed in detail. This approach has been successfully applied for genome editing in crop plants: camelina, cotton, lemon, melon, orange, peanut, rice, soybean, and wheat. Overall, this study underscores the importance of *in-planta* methodologies in overcoming the limitations of tissue culture and advancing the field of plant genome editing.

## 1 Introduction

Plant genetic engineering and genome editing will play an increasingly important role in future food security (Simkin, 2019; Varshney et al., 2021). Plant transformation is essential for functional genetic studies; it facilitates the discovery of biological processes and traits, which biotechnology could then use as a guide to improve crop genetics (Anjanappa and Gruissem, 2021). CRISPR/Cas9 technology is currently the most employed genome editing (GE) tool in crop breeding (Cardi et al., 2023). It is more efficient, precise, more accessible to apply, and cheaper when compared to other GE technologies, such as zinc finger nucleases (ZFNs) and transcription activator-like effector nucleases (TALENs) (Afzal et al., 2020). CRISPR/Cas9 could fundamentally improve crop breeding by allowing breeders to access and incorporate large amounts of genetic diversity stored in wild species into their breeding programs (Wolter et al., 2019). The original CRISPR/Cas9 system creates high-precision double-strand breaks (DSB) in DNA based on a single-guide RNA (sgRNA) complementary to the genomic target site. The enzymatic active sgRNA-Cas9 ribonucleoprotein complex recognizes a short sequence following the target site named protospacer adjacent motif or PAM. Once PAM is recognized, Cas9 activates and cuts the DNA (Jinek et al., 2012). The most widely employed and efficient method for delivering the CRISPR/Cas9 components in plants is the stable integration or transitory expression of the foreign DNA by *Agrobacterium*-mediated genetic transformation and plant regeneration through *in vitro* tissue culture methods (Zhang et al., 2021). Plant genetic transformation has advanced rapidly over the past 25 years. However, developing complimentary tissue culture methods remains a significant constraint to plant transformation.

Developing tissue culture techniques and methods is a complex process that involves optimizing several factors at each stage. These stages include the isolation of cells or specialized tissues, the growth of those cells or tissues under defined aseptic conditions, the establishment of transformation protocols with *Agrobacterium spp.* or other methods (biolistic delivery, polyethylene glycol, or electroporation), and the regeneration of the transformed plant. Therefore, genetic transformation and plant regeneration through tissue culture are both time-consuming and labor-intensive processes. For example, the transformation of rice or tomato can take between 6 and 12 months, even following established protocols (Kim, 2020). Another issue when employing tissue culture methods may arise is somaclonal variation, meaning unwanted variations for researchers who require genetic fidelity in their clones. Plant lines obtained through CRISPR/Cas9 genome editing could carry genetic variability or genetic rearrangements independent from the editing *per se* (Arencibia et al., 2019).

*In-planta* methods are alternative plant transformation methods with the potential to overcome the limitations and disadvantages of tissue culture. The advantages of *in-planta* transformation further widen the possibilities and applications of plant genetic engineering technologies. This review discusses strategies developed for CRISPR/Cas9 genome editing through *in-planta* transformation methods focusing on crops and recently developed plant genome editing techniques that bypass tissue culture steps.

The public database PubMed^®^ was searched for peer-reviewed articles that contained keywords: “*in-planta*” and “CRISPR.” Articles included in this study must have been published between January 2012 and October 2024. A total of 243 articles met the search criteria and were manually filtered. The articles’ titles, abstracts, and methodology were read and analyzed to select the studies limited to using CRISPR/Cas9 genome editing implemented *in-planta* transformation methods. The search and subsequent manual filtering produced 64 papers, from which 52 were studied with model plants, and 12 were with crop plants. Additionally, specific searches on Google Scholar for supporting studies were performed.

## 2 What is *in-planta* transformation?

The concept of simplifying the genetic transformation of plants has been explored for 40 years. Some of the oldest documented transformation protocols that can be categorized as *in-planta* describe the techniques: pollen-tube mediated pathway in cotton (Zhou et al., 1983) and rice (Luo and Wu, 1989), embryo *Agrobacterium*-mediated infection in maize (Graves and Goldman, 1986) and soybean (Chee et al., 1989), and floral dip in Arabidopsis (Clough and Bent, 1998). Many more techniques have been developed for several plant species (Kaur and Devi, 2019; Zlobin et al., 2020; Saifi et al., 2020).

The *in-planta* transformation methods can be categorized under the definition of means of plant genetic transformation with no or minimal tissue culture steps meeting the conditions for minimal steps: short duration with a limited number of transfers, high technical simplicity, limited list of hormones, and that the regeneration does not undergo callus development (Bélanger et al., 2024). This recently proposed definition for the *in-planta* transformation methods englobes the multiple protocols developed over the years aiming to simplify the genetic transformation of plants. At the same time, it sets a line to separate some protocols that the authors might consider *in-planta* but still rely on *in vitro* tissue culture steps or extensive use of hormones. Bélanger et al. (2024) have recently compiled and contextualized the significance of the comprehensive array of *in-planta* transformation techniques. Our review distinguishes itself by focusing exclusively on articles that adhere to stringent criteria for qualifying as accurate in-planta transformation protocols, offering a refined perspective on this rapidly evolving field. Accurate protocols, in this context, are defined as reliable, reproducible, and consistent in achieving successful transformation outcomes while adhering to a minimalistic approach. Specifically, accurate in-planta transformation methods involve plant genetic transformation approaches with minimal or no tissue culture steps. These protocols meet the following criteria: they require a short duration with limited transfers, demonstrate high technical simplicity, utilize a minimal list of hormones, and achieve regeneration without callus development. Articles conforming to this definition were chosen to ensure the inclusion of methodologies that exemplify these rigorous standards.

*In-planta* transformation methods hold the potential to be less labor-intensive, less time-consuming, and cheaper than traditional methods like calli transformation and regeneration (Saifi et al., 2020). Nevertheless, the differences between *in-planta* and *in vitro* transformation methods should be addressed case-specific, as *in vitro*-dependent transformation methods are well developed and established for some species.

**FIGURE 1:**
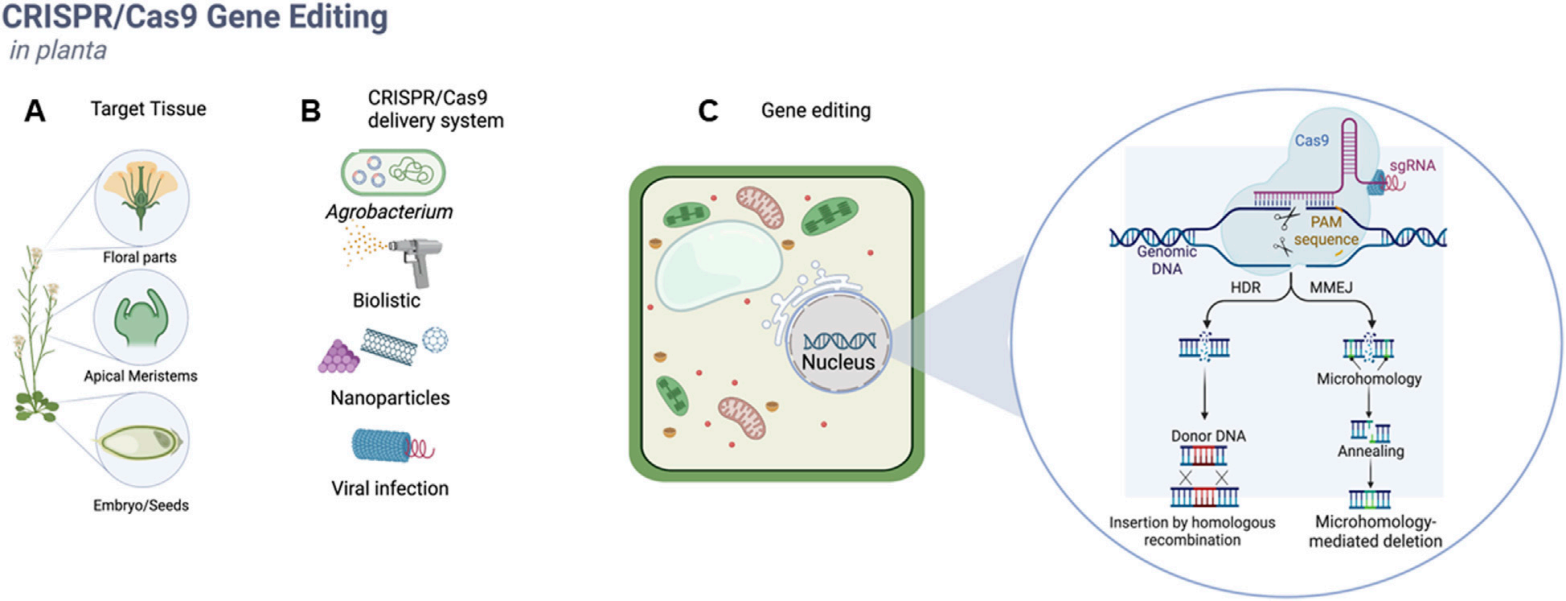
In-planta CRISPR/Cas9 gene-editing strategies methods can be divided according to the target tissue selected for transformation **(A)**, strategies for the delivery of CRISPR/Cas9 components inside the plant cell **(B)** or applications such as gene editing, gene targeting, gene regulation **(C)**.

A practical approach to study strategies for achieving *in-planta* transformation is to categorize them according to the type of explant target (meristematic tissues, floral parts, and embryos) (Figure 1A) and transformation methods (*Agrobacterium*, biolistic, nanoparticles, viral infection) employed to introduce the CRISPR/Cas9 components inside the plant cell.

## 3 Bibliometric analysis

Analyzing publication trends provides a structured view of research activity, revealing predominant topics and methodological advancements in genome editing and *in-plant* transformation. Applied to plant biotechnology and genetic transformation, this approach highlights the primary focus areas, targeted tissues, delivery methods, and critical terms frequently emphasized across studies.

**FIGURE 2:**
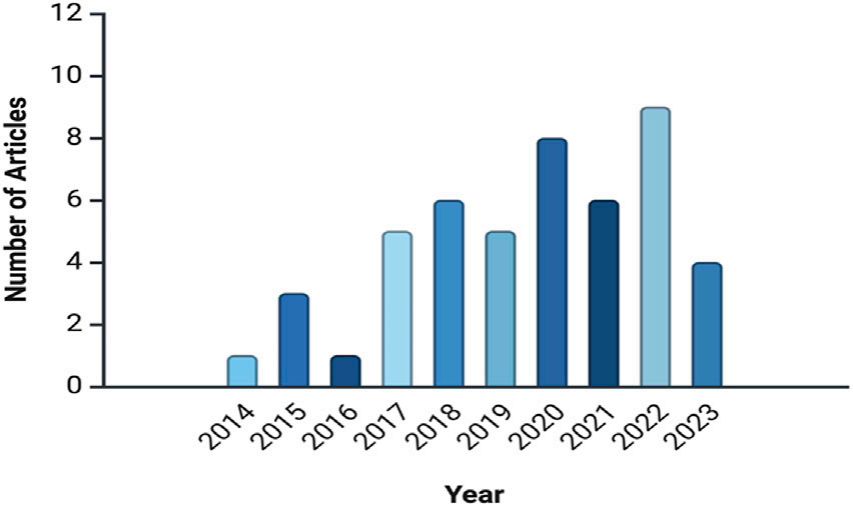
Annual publication trends in *in-planta* genome editing research, indicate a rise in interest from 2014 to 2023. This analysis is based on data from PubMed, as described in the text.

Figure 2 illustrates the number of articles published annually, indicating an increasing interest in *in-planta* genome editing research over time. The number of publications rose from 1 article in 2014 to peaks of 7 articles in 2017 and 2018, followed by a high of 8 articles in 2020 and 9 articles in 2022. This upward trend suggests a growing research focus, likely driven by advancements in genome-editing technologies or emerging agricultural challenges.

**FIGURE 3:**
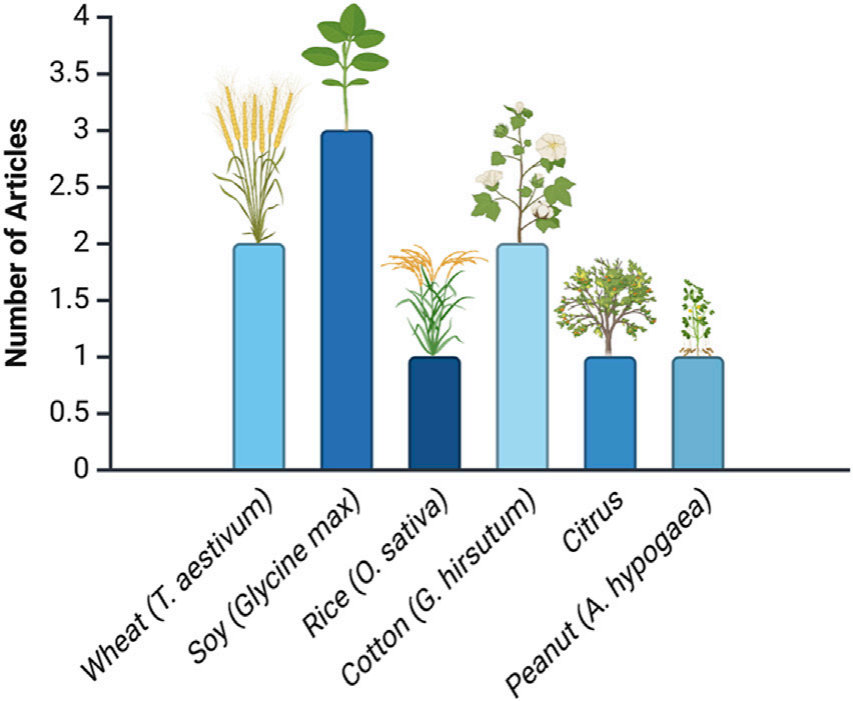
Distribution of research articles across crop species. This analysis is based on data from PubMed, as described in the text.

Figure 3 shows the distribution of research articles across various crop species. With three articles, soybean (*Glycine max*) is the most prominent crop, reflecting its critical role in agriculture due to its economic importance and specific agronomic challenges, such as pest resistance and yield enhancement. In contrast, crops like wheat (*Triticum aestivum*), cotton (*Gossypium* spp.), rice (*Oryza sativa*), citrus (*Citrus* spp.), and peanut (*Arachis hypogaea*) have only one or two articles each.

**FIGURE 4:**
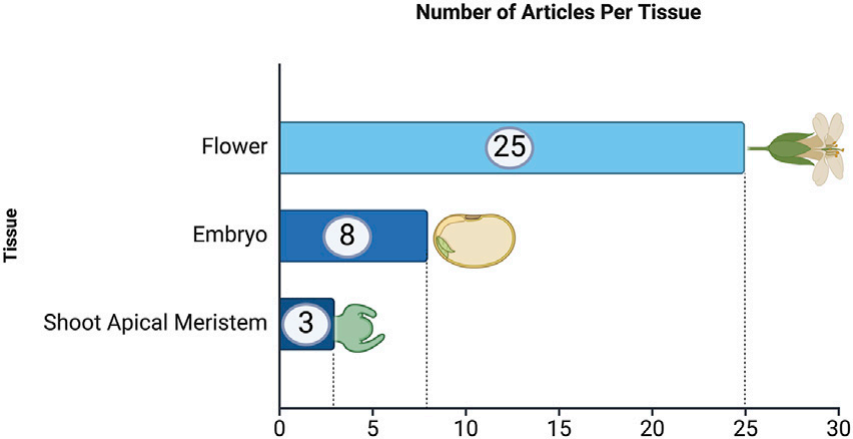
Frequency of articles by plant tissue type. This analysis is based on data from PubMed, as described in the text.

Regarding the number of articles per tissue, Figure 4 shows the distribution of articles focused on different tissue types used in *in-planta* transformation studies. Flowers are the most frequently used tissue, with 25 articles dedicated to this approach. Embryos are the second most studied tissue type, with 8 articles. Lastly, shoot apical meristems have been used in 3 articles, representing a smaller but significant focus within *in-planta* studies.

**FIGURE 5:**
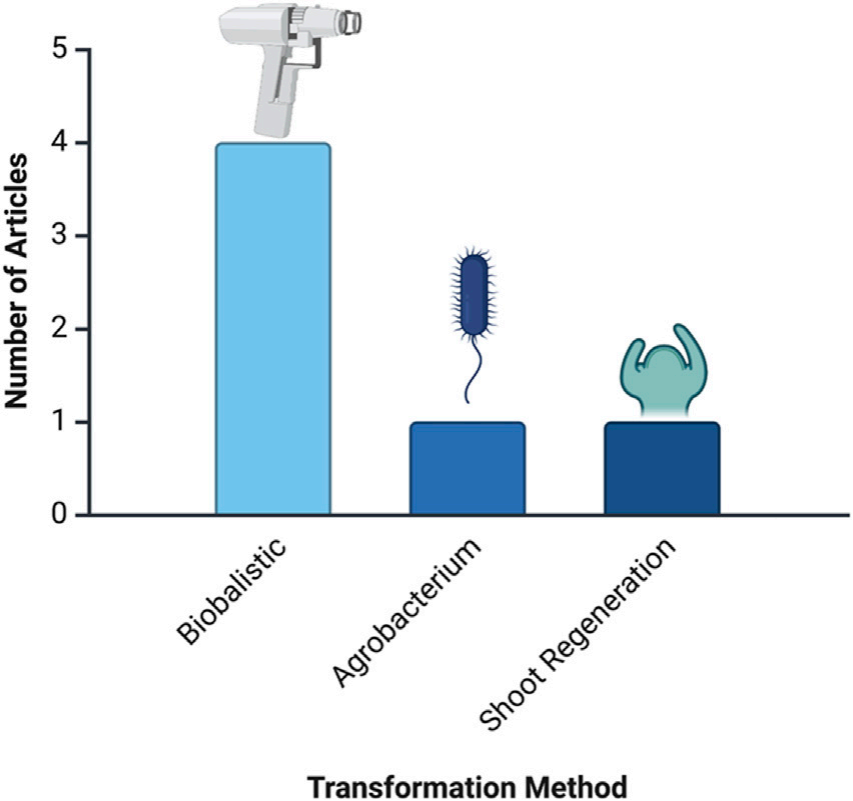
Number of articles by genetic transformation delivery method. This analysis is based on data from PubMed, as described in the text.

Figure 5 highlights the number of articles per delivery method, showcasing the various genetic transformation methods used across studies. *Agrobacterium*-mediated transformation is the most prevalent, with 35 articles underscoring its established role as a reliable delivery mechanism for plant genetic material. Other methods, such as floral dip, biolistic, and agroinfiltration, are used less frequently, indicating their application in specific cases or as alternatives to *Agrobacterium*. Less common methods, including PEG, microneedles, and carbon nanotubes, appear in only one article each, suggesting their experimental nature or niche applications in plant biotechnology.

**FIGURE 6:**
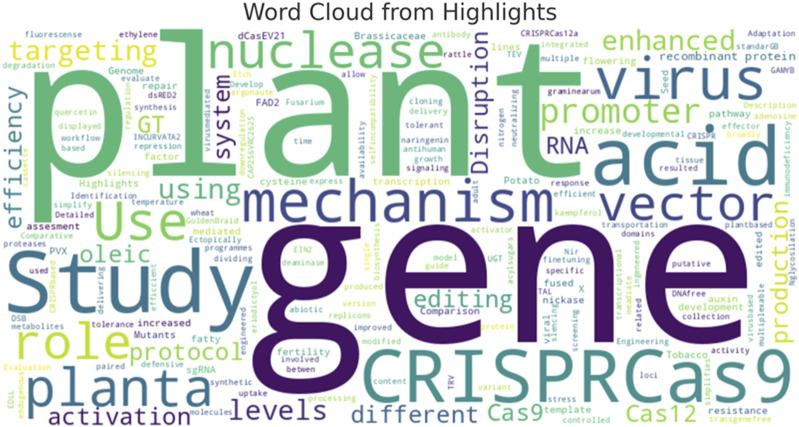
Key terms and concepts in plant genome editing research. This analysis is based on data from PubMed, as described in the text.

Regarding the key terms and concepts emphasized in the analyzed studies, Figure 6 reveals prominent terms such as “gene,” “plant,” “CRISPR/Cas9,” and “nuclease,” underscoring a solid focus on gene editing and molecular biology in plant research. Terms like “vector,” “mechanism,” and “promoter” highlight critical areas of interest in the delivery and regulation of genetic modifications. Additionally, terms such as “virus,” “enhanced,” and “efficiency” suggest ongoing efforts to optimize gene-editing methods and investigate various vectors for transformation.

## 4 Foreign DNA *in-planta* delivery methods

*In-planta* genetic transformation methods, including *Agrobacterium*-mediated transformation, biolistic delivery, and electroporation, are essential for introducing foreign DNA into plant cells. Each employs distinct mechanisms and exhibits varying applicability. *Agrobacterium*-mediated transformation utilizes *A. tumefaciens*, which effectively transfers T-DNA from its Ti plasmid into the cells of dicotyledons, leveraging the host’s DNA repair machinery for integration. This process is facilitated by virulent proteins that promote the incorporation of foreign DNA into the plant genome (Gelvin, 2017).

Biolistic delivery, commonly called the gene gun method, propels DNA-coated microparticles—typically composed of gold or tungsten—into plant tissues using high-pressure gas. This technique demonstrates a broader host range applicability, particularly for recalcitrant species, including monocots; however, it may cause cellular damage and lead to multiple gene insertions due to the random incorporation of DNA into the plant genome upon penetration of the cell wall and membrane (Altpeter et al., 2005).

Electroporation involves applying electrical pulses to protoplasts, temporarily permeabilizing cell membranes and facilitating DNA uptake. This technique capitalizes on the plant’s natural repair processes for genomic integration. Nonetheless, a significant challenge associated with electroporation is regenerating viable plants from the transformed protoplasts (Bates et al., 1988; Joersbo and Brunstedt 1991).

These transformation techniques have unique advantages and limitations, influencing their effectiveness for specific plant species and transformation objectives. Thus, they contribute significantly to advancing plant biotechnology through their diverse DNA integration mechanisms.

## 5 Explant types for *in-planta* transformation

*In-planta* genetic transformation using meristematic tissues, floral parts, and embryos offers alternative routes to traditional tissue culture methods by targeting specific plant structures for genetic modification. Meristematic tissues are highly active regions where cell division occurs, such as shoot apical meristems (SAM), which can be directly transformed using *A. tumefaciens* or biolistic methods. This allows for integrating foreign DNA into the actively dividing cells, leading to the generation of transformed leaves, stems, or flowers. However, meristematic transformation often results in chimeric plants, as not all cells are uniformly transformed, requiring progeny screening to isolate fully edited lines.

Floral part transformation involves targeting germline cells within the reproductive organs to ensure heritable modifications. The most well-known technique is the floral dip method, where entire inflorescences are immersed in an *Agrobacterium* suspension, enabling the bacteria to penetrate floral tissues and deliver genetic material to the germline. This method, particularly effective in *Arabidopsis thaliana*, is low-cost, bypasses tissue culture, and facilitates high-throughput transformation, although its efficiency is species-dependent.

Embryo transformation focuses on the genetic modification of embryos during seed germination. Techniques such as *Agrobacterium*-mediated infection or biolistic bombardment of embryos enable the direct delivery of CRISPR/Cas9 components or other transgenes to cells destined to become meristems or other vital tissues. This approach is advantageous for recalcitrant species where traditional tissue culture-based transformations are inefficient, allowing for stable and potentially genotype-independent transformations.

### 5.1 Genetic transformation of floral parts

Floral tissues are a suitable target for *in-planta* transformation because flower germline cells’ (pollen and ovules) efficient transformation will pass through progeny after fertilization (Bent, 2006). The pollen tube pathway method delivers foreign DNA into an ovule as fertilization occurs, avoiding the egg cell layers that prevent direct contact with DNA or *Agrobacterium* (Kaur and Devi, 2019). This method requires that a foreign DNA solution be applied by drop or microinjection onto the style of the recipient tissue; then, the DNA is transported via pollen tube growth to the ovary. The integration occurs in the early stages of embryo or even zygote formation (Ali et al., 2015). The pollen tube transformation method was first reported in cotton by Zhou et al. (1983) and nowadays is still used as an alternative to a cotton genotype-independent transformation method (Wang et al., 2019).

Another *in-planta* transformation method is the floral dip, which immerses the complete inflorescence in a solution of *Agrobacterium tumefaciens* containing a strong surfactant (Clough and Bent, 1998). Seeds are then collected, germinated, and selected for transgenics. An efficient surfactant is critical for floral dip success, and it improves *Agrobacterium* cell penetration into floral tissues (Clough and Bent, 1998). Some commonly used surfactants for plant genetic transformation are Silwett-L-77, Tween-20, and Triton X100 (Kim et al., 2009). The floral dip method requires minimal labor, is less time-consuming, and avoids using plant tissue culture. Its advantages allow high throughput transformation with frequencies up to 3% in *Arabidopsis thaliana,* and it has become the method of choice for transforming this model organism (Bent, 2006). Studies of floral dip transformation, which confirmed efficient transformation by molecular or protein expression and inheritance, have been reported, although less widely, in crops such as maize (Mamontova et al., 2010; Guiqin et al., 2012), wheat (Zale et al., 2009; Singh and Kumar, 2022), tomato (Yasmeen et al., 2009), soybean (Liu et al., 2009), and camelina (Liu et al., 2012).

Other approaches to floral tissue transformation, in addition to dipping, include spraying, inoculating, dripping, and injecting *Agrobacterium* suspension directly into female floral reproductive tissues (Kaur and Devi, 2019).

### 5.2 Genetic transformation of shoot apical meristems

Another common *in-planta* transformation technique is to apply *A. tumefaciens* suspension into meristematic tissue. Transformation of shoot apical meristems (SAM) produces transformed structures such as leaves, stems, and flowers. The *in-planta* SAM transformation method has been used in crops such as cotton, sunflower, and field beans (Kesiraju and Sreevathsa, 2017).

The transformation of meristematic tissues can be done through methods other than *Agrobacterium,* such as biolistic ones. *In-planta* biolistic meristematic tissue transformation has been done in various crops, including wheat (Imai et al., 2020), cowpea (Ivo et al., 2008), and soybean (Paes de Melo et al., 2020). One disadvantage of SAM transformation methods is the production of chimeric transgenic plants since it is practically impossible to transform every cell within meristems. This constraint is usually overcome by screening the progeny of transformed plants for successfully transformed events.

### 5.3 Genetic transformation of embryos

The first reported *in-planta* transformation method was cultivating germinating seeds with *Agrobacterium* (Feldmann and Marks, 1987). Stable transgenic *Arabidopsis* plants resistant to the antibiotic G418 were obtained by transferring a vector with the *nptII* selection marker gene. Since then, *in-planta* transformation has evolved and improved (Feldmann and Marks, 1987).

Embryos can be exposed to bacteria alone or attached to the cotyledon. This method aimed to transform the apical meristematic tissue during germination by direct imbibition or injury. This method has the potential to be genotype-independent, as seen in species like cotton (Kesiraju et al., 2020), pigeon pea (Sankara-Rao et al., 2008), bell pepper (Manoj-Kumar et al., 2011), and peanut (Keshavareddy et al., 2013). An attribute that is a significant advantage when attempting to transform typically recalcitrant crop varieties.

The most common embryo transformation method consists of pricking meristematic tissue with a sterile needle before, during, or shortly after germination and then dipping the tissue in an *Agrobacterium* solution for about 40 min (Supartana et al., 2005). This method has been used in a wide variety of plants, including essential cash crops such as rice (Supartana et al., 2005), wheat (Razzaq et al., 2011), flax (Kesiraju et al., 2021), and tomato (Shah et al., 2015). Paes de Melo et al. (2020) developed a novel strategy for another *in-planta* soybean transformation method using biolistic to injure the embryos and *A. tumefaciens* for DNA delivery. Although they achieved a similar regeneration and transformation efficiency (9.8%) compared to traditional methods, they report a more cost-effective and straightforward method without any detectable chimeras.

## 6 *In-planta* CRISPR/Cas9 genome editing in model plants

Research utilizing the model plants *A. thaliana* and *N. benthamiana* predominantly centers on enhancing the efficiency of CRISPR/Cas9 gene editing (Table 1). A significant focus has been optimizing novel CRISPR-associated proteins, including paired nickases and variants of Cas9, to improve gene targeting and regulation. Numerous protocols have been established to evaluate the efficacy of different promoters, nucleases, and donor DNA template delivery strategies, thereby refining the overall gene editing process.

**TABLE 1:** Overview of target genes, applications, and advances in protocol improvement, molecular studies, and plant enhancement using CRISPR/Cas9-mediated gene editing methods in *Arabidopsis thaliana* and *Nicotiana benthamiana* across different studies.

Plant specie	Transformation method	Target gene	Application	Highlights	Reference
*Arabidopsis thaliana*	Floral dip	ADH1 Locus	Protocol improvement	CRISPR-Cas9 mediated in planta GT by paired nickases	Schiml et al. (2014)
		LUC-based reporter gene	Protocol improvement	Comparative assesment of Cas9 nucleases efficiency	Johnson et al. (2015)
		FT	Protocol improvement	Use of dividing tissue specific promoter (*INCURVATA2*)	Hyun et al. (2015)
		Not specified	Protocol	CRISPR-Cas9 mediated in planta GT protocol	Schiml et al. (2017)
		Als	Protocol improvement	Evaluation of different developmental controlled promoters and modified nucleases	Wolter et al. (2018)
		PDS3	Protocol develpment	Use of Cas9 nickase fused to adenosine deaminase	Kang et al. (2018)
		PPO	Protocol development	Ectopically integrated repair template and CRISPR-Cas9 DSB for gene targeting	de Pater et al. (2018)
		gl1	Protocol improvement	Comparison of repair template availability betwen viral replicons and in planta gene targeting	Hahn et al. (2018)
		Als	Protocol improvement	Enhanced GT using CRISPR/Cas12a nuclease	Wolter and Puchta. (2019)
		IAMT	Protocol improvement	Identification of transgene-free edited plants by fluorescense	Aliaga-Franco et al. (2019)
		Als	Protocol	Use of temperature tolerant version of Cas12 nuclease	Merker et al. (2020a)
		PDS	Protocol improvement	Comparison of different promoters efficiency	Wolabu et al. (2020)
		Als	Protocol improvement	Gene targeting efficiency enhanced by Cas12 nuclease variant	Merker et al. (2020b)
		UGT	Molecular study	Study on UGT role in abiotic stress tolerance mechanism	Li et al. (2017)
		AP2M	Molecular study	Study on the mechanism of self-incompatibility response in Brassicaceae	Yamamoto et al. (2018)
		NiR	Molecular study	Description of the role of Nir gene in nitrogen uptake and plant growth	Costa-Broseta et al. (2020)
		FAD2	Plant enhacement	Disruption of the gene FAD2 increases the levels of oleic acid	Park et al. (2021)
		EIN2	Pathogen resistance	Disruption of gene EIN2 resulted in downregulation of the ethylene signaling pathway. Mutants displayed enhanced *Fusarium graminearum* resistance	Low et al. (2022)
		MYB transcriptional factor	Molecular study	Study of the role of the wheat GAMYB transcriptional factor in flowering time and fertility	Yang et al. (2023)
		GL1	Protocol improvement	Use of dsRED2 cassette to simplify the screening of putative edited plants	Kong et al. (2023)
		AUX1, PIN3, and TAA1	Molecular study	Study of the role of auxin synthesis and auxin transportation related genes in fertility	Tan et al. (2023)
*Nicotiana benthamiana*	Agroinfiltration	Bs3 promoter	Proof of concept	Protocol development for gene transcription activation and repression using fused Cas9 with EDLL and TAL effector domains	Piatek et al. (2015)
		XT1 and XT2	Protocol improvement	Adaptation of CRISPR/cas9 cloning workflow to GoldenBraid standar(GB)	Vazquez-Vilar et al. (2016)
		PDS3	Protocol improvement	Assesment of Cas12 activity in different loci and model plants	Bernabé-Orts et al. (2019)
		GFP	Protocol development	Engineering of a plant RNA virus-based vector for DNA-free in planta delivery of CRISPR–Cas9	Ma et al. (2020)
		GFP	Molecular study	Study of the mechanism of RNA single guide processing in planta	Cody and Scholthof. (2020)
		B-galactosidase genes	Molecular study	Study the mechanism of N-glycosilation in plants for the production of recombinant proteins	Kriechbaum et al. (2020)
		12 different flavonoid genes	Metabolic engineering	Develop of efficient activation programmes for the metabolites naringenin, eriodictyol, kaempferol, and quercetin meadiate by multiplexable CRISPR activator dCasEV2.1	Selma et al. (2022a)
		NbVPE-1a, NbVPE-1b, and NbCysP6	Molecular farming	Disruption of plant cysteine proteases involved in the degradation of the plant-based produced anti-human immunodeficiency virus broadly neutralizing antibody, CAP256-VRC26.25	Singh et al. (2022)
		proximal elements within synthetic promoters	Protocol development/Synthetic Biology	Use of dCasEV2.1 activation system to evaluate a collection of synthetic promoters and its fine-tuning	Moreno-Gimenez et al. (2022)
	Leaf disc	AGO1	Molecular study	Study of mechanism of argonaute proteins in RNA slencing pathways	Ludman and Fátyol. (2021)
		RDR6	Plant enhancement	Recombinant protein production in plants	Matsuo and Atsumi. (2019)
		NtALS	Molecular study	Mechanism of biosynthesis of plant defensive molecules acylsugars	Chang et al. (2020)
	Virus-Induced	NbFT, NbPDS3 and NbXT2B	Protocol development	The *Potato virus X vector* (PVX) was ingeneered to express multiple sgRNA and allow efficcient gene editing in adult plants	Uranga et al. (2021a)
		NbXT1, NbFT	Protocol development	Genome editing using the viral vectors Tobacco Etch Virus (TEV) and Potato virus X (PVX)	Uranga et al. (2021b)
		PDS	Protocol development and recombinant protein production	An improved vector system based on tobacco rattle virus (TRV) that simplifies gene silencing, also used for recombinant protein production	Aragonés et al. (2022)
		MYB transcriptional factors (TFs)	Metabolic engineering	Development of a CRISPR-based endogenous gene regulation system by delivering sgRNA targeting transcription factors using engineered plant virus vectors	Selma et al. (2022b)
		Not specified	Protocol	Detailed protocol for virus-mediated CRISPR/Cas9 genome editing	Uranga et al. (2023)

In *A. thaliana*, specific targets for CRISPR/Cas9-mediated gene editing include key loci, such as *ADH1* and *PDS3*, alongside reporter genes like *LUC*. These studies have explored the roles of various genes in critical physiological processes, including cell signaling pathways, nitrogen uptake, and mechanisms conferring tolerance to abiotic stresses. Notably, gene disruption efforts, such as the inactivation of *FAD2*, have demonstrated increased oleic acid levels. In contrast, the disruption of *EIN2* has led to enhanced resistance to *Fusarium graminearum* through the downregulation of the ethylene signaling pathway (Table 1). The delivery of the CRISPR/Cas9 components through the floral dip transformation method is the most used strategy for the genome editing of *A. thaliana* (Zlobin et al., 2020). Previous studies of CRISPR/Cas9 genome editing in Arabidopsis show low mutation frequency and heritability, possibly due to plasmid constructs with strong promoters with low activity in germ-line cells (Khumsupan et al., 2019). However, researchers have implemented tissue-specific promoters active in ovules, zygotes, and early embryos to overcome this issue (Zlobin et al., 2020).

In *N. benthamiana*, innovative applications of agroinfiltration have emerged, demonstrating the potential of using fused Cas9 with EDLL and TAL effector domains for gene transcription activation and repression. Establishing various protocols has enabled the assessment of Cas12 activity, the development of RNA virus-based vectors for DNA-free in planta delivery of CRISPR/Cas9, and investigations into RNA processing and glycosylation mechanisms. Additionally, metabolic engineering initiatives have focused on efficiently activating multiple flavonoid genes, while the disruption of cysteine proteases has been explored for molecular farming applications (Table 1).

Furthermore, virus-induced methods utilizing engineered viral vectors, such as Potato virus X (PVX) and Tobacco Etch Virus (TEV), have been developed to facilitate efficient gene editing in adult plants.

This review is concentrated on crop plants, and as such, model plant studies will only be discussed further after being compiled and classified according to the transformation methods employed (Table 1). Nevertheless, the advancements in these studies underscore a comprehensive approach to optimizing CRISPR technologies, demonstrating their wide-ranging applicability in molecular studies, metabolic engineering, plant synthetic biology, and molecular farming. Ultimately, these efforts contribute significantly to the progress of plant biotechnology.

## 7 *In-planta* CRISPR/Cas9 genome editing in agronomically essential crops

*In-planta* transformation methods have been used to produce genetically modified organisms (GMOs) and in functional genetics studies for a wide variety of crops (Saifi et al., 2020). However, studies exploiting the advantages of *in-planta* transformation methods for delivering CRISPR/Cas9 components into the plant cells are limited to a few essential crop species: camelina, citrus, cotton, melon, peanut, rice, soybean, and wheat. These are detailed below.

### 7.1 Camelina

*Camelina sativa* was found to be the only non-model plant in which *in-planta* transformation methods have been used widely for CRISPR-based genome editing by different research groups. The advances and challenges of *C. sativa* genome editing have been reviewed previously (Zlobin et al., 2020; Ghidoli et al., 2023).

The floral dip transformation method, coupled with the CRISPR/Cas9 system for gene knockout, is the standard approach in the studies conducted on *Camelina sativa* (Table 2). The primary aim of CRISPR-based genome editing in this species has been to enhance seed oil composition (Jiang et al., 2017; Morineau et al., 2017; Lee et al., 2021; Aznar-Moreno and Durrett, 2017; Ozseyhan et al., 2018; Han et al., 2022). Other applications include examining interactions with fungal pathogens (Darvishi et al., 2021), improving seed protein quality (Lyzenga et al., 2019), inducing early flowering stages (Bellec et al., 2022), and developing glucosinolate-free seeds (Hölzl et al., 2023).

**TABLE 2:** Summary of transformation methods targeting various genes for improving seed oil composition, plant development traits, and disease resistance.

Transformation method	Target gene	Application	Highlights	Reference
Floral dip	Fatty acid desaturase 2 (FAD2)	Seed oil composition improvement	Oleic acid content was increased from 16% to over 50% of the fatty acid composition	Jiang et al. (2017)
Floral dip	Fatty acid desaturase 2 (FAD2)	Seed oil composition improvement	Camelina lines with various lipid profiles, ranging from 10% to 62% oleic acid accumulation in the oil	Morineau et al. (2017)
Floral dip	Fatty acid desaturase 2 (FAD2)	Seed oil composition improvement	Knockout of all three pairs of FAD2 homoeologs led to a stunted bushy phenotype, but greatly enhanced MUFA levels (by 80%) in seeds	Lee et al. (2021)
Floral dip	CRUCIFERIN C (CsCRUC)	Seed protein composition improvement	Seed with increase proportion of alanine, cysteine and proline, and decrease of isoleucine, tyrosine and valine	Lyzenga et al. (2019)
Floral dip	Diacylglycerol acyltransferase *(DGAT1)* and phospholipid: diacylglycerol acyltransferase 1 *(PDAT1)*	Seed oil composition improvement	Lines with higher levels of linoleic acid (18:2) instead of linolenic acid	Aznar-Moreno and Durrett. (2017)
Floral dip	Fatty acid elongase 1 (*FAE1*)	Seed oil composition improvement	Very long-chain fatty acids VLCFAs were reduced to less than 2% compared to over 22% in the wild type	Ozseyhan et al. (2018)
Floral dip	Fatty acid elongase 1 (*FAE1*)	Seed oil composition improvement	Increased levels of EPA, DHA and other omega-3 LC-PUFAs in	Han et al. (2022)
Floral dip	Cinnamoyl-CoA Reductase 4 (CsCCR4)	Molecular study	Study of the role of CsCCR4 gene in the fungal *S. sclerotiorum* resistance	Darvishi et al. (2021)
Floral dip	FLOWERING LOCUS C (FLC), SHORT VEGETATIVE PHASE (SVP), LIKE HETEROCHROMATIN PROTEIN 1 (LHP1), TERMINAL FLOWER 1 (TFL1), and EARLY FLOWERING LOCUS 3 (ELF3)	Plant development trait improvement	Certain mutants showed stable early-flowering trait after five generations also presented: determinate flowering, shorter stature and/or basal branching	Bellec et al. (2022)
Floral dip	Glucosinolate transporter 1 and 2 (GTR1-GTR2), and transcription factors MYB28, MYB29	Plant enhancement	Complete loss of glucosinolates, representing the first glucosinolate-free *Brassicaceae* crop	Hölzl et al. (2023)

A significant focus has been on the Fatty Acid Desaturase 2 (*FAD2*) gene, which is essential for modifying oil composition. Research by Jiang et al. (2017) demonstrated that transforming *Camelina* lines could increase oleic acid content from 16% to over 50% of the total fatty acid profile. Morineau et al. (2017) reported oleic acid accumulation levels ranging from 10% to 62% in newly developed lines. Lee et al. (2021) further revealed that knocking out all three pairs of *FAD2* homeologs led to a stunted bushy phenotype while significantly boosting monounsaturated fatty acid (MUFA) levels in seeds by 80%.

In addition to *FAD2*, the Cinnamoyl-CoA Reductase 4 (*CsCCR4*) gene has been investigated for its role in enhancing resistance to the fungal pathogen *Sclerotinia sclerotiorum* (Darvishi et al., 2021). The genes Diacylglycerol Acyltransferase (*DGAT1*) and Phospholipid: Diacylglycerol Acyltransferase 1 (*PDAT1*) have also been targeted for improvements in oil composition, with Aznar-Moreno and Durrett (2017) noting increased linoleic acid (18:2) levels, which shifts the fatty acid profile away from linolenic acid.

Manipulation of the Fatty Acid Elongase 1 (*FAE1*) gene has resulted in notable changes; Ozseyhan et al. (2018) found that very long-chain fatty acids (VLCFAs) were reduced to less than 2%, compared to over 22% in wild-type plants. Han et al. (2022) reported increases in eicosapentaenoic acid (EPA), docosahexaenoic acid (DHA), and other omega-3 long-chain polyunsaturated fatty acids (LC-PUFAs) in transformed lines.

Research on plant developmental traits has targeted genes such as Flowering Locus C (*Flc*), Short Vegetative Phase (*Svp*), Like Heterochromatin Protein 1 (*Lhp1*), Terminal Flower 1 (*TFL1*), and Early Flowering Locus 3 (*ELF3*). Bellec et al. (2022) noted that specific mutants exhibited stable early-flowering traits after five generations, along with determinate flowering, shorter stature, and basal branching. Additionally, investigations into glucosinolate transporters (GTR1-GTR2) and transcription factors (MYB28, MYB29) led to the complete loss of glucosinolates, resulting in the development of the first glucosinolate-free Brassicaceae crop (Hölzl et al., 2023).

These findings highlight the efficacy of the floral dip transformation method in advancing *Camelina* crop improvement, particularly in oil quality and plant resilience to stressors, while demonstrating the potential benefits of CRISPR technologies in agricultural practices.

### 7.2 Citrus

Citrus is a major fruit crop globally, but various biotic and abiotic stresses often compromise its productivity and survival. Transgenic approaches have been successfully used to develop genetic resistance against several pests and diseases (Alquézar et al., 2022). Building on these advancements, genome editing technologies like CRISPR/Cas9 offer a more precise and efficient alternative, enabling targeted genetic modifications without introducing foreign DNA.

In this context, the study by Khadgi et al. (2024) presents six *in-planta* transformation protocols for citrus epicotyls and auxiliary meristems, focusing on their shoot regeneration efficiency. Among these, two protocols stood out for their high success rates: the blunt cut with tip inoculation (85%) and the apical bud incision method, which involved growing auxiliary meristems for 3–5 days, followed by fresh micro wounds and cotton inoculation (95%). Briefly, the blunt cut with tip inoculation consists of cutting off shoots of 4–6 weeks seedlings, leaving 3–4 cm of epicotyls, and covering them with a small pipet tip (10 µL) filled with *Agrobacterium* inoculation solution for 1 h. The second promising *in-planta* protocol consists of cutting off the shoot of 4–6 weeks seedlings, leaving auxiliary meristems, and allowing them to grow for 3–5 days. Then, a crosscut on the epicotyl and three wounds on each meristem were made using a fine needle. The epicotyls were immediately covered with small cotton balls saturated with *Agrobacterium* solution and incubated for 2 days. Kanamycin-soaked cotton balls were used to eliminate the *Agrobacterium* solution. After 2 weeks of dark incubation, all the seedlings were transferred to light conditions, and the wounded sites produced many new shoots. The shoots were selected using the GFP visual marker, and after 1.5 months of growth, they were analyzed for putative genome editing. The developed *in-planta* transformation protocols were used to deliver the CRISPR/Cas9 system targeting the genes *SWEET10*, *SWEET12*, and *SWEET15* in Limoneira 8A Lisbon lemon and Pineapple sweet orange. They obtained 11 transgenic lines of lemon and three transgenic lines of orange. PCR analysis and Sanger sequencing confirmed the mutations in the targeted genes with most seedlings showing high knockout scores (over 90%) for at least one of the two sgRNAs. This confirms the successful gene editing of all the 14 transgenic lines obtained from the previously described methods (Khadgi et al., 2024). This study is a clear example of the potential of *in-planta* transformation protocols for advancing plant breeding in species recalcitrant to tissue culture.

Although the study by Alquezar et al. (2022) does not strictly fit the definition of an *in-planta* transformation protocol as outlined by Bélanger et al. (2024), it presents a promising method for DNA-free delivery in planta. This research highlights the importance of selection marker genes for the efficient recovery of transgenic citrus plants. The authors demonstrated that mutated forms of the acetolactate synthase (*ALS*) gene when combined with the herbicide imazapyr (IMZ) as a selection agent, enable the development of cisgenic regenerants—plants that lack the bacterial genes commonly used in transgenic selection. Additionally, this approach allows for the generation of edited, non-transgenic plants with modified endogenous *ALS* genes that confer resistance to IMZ. In their study, citrus mutants exhibiting IMZ-resistant *ALS* forms were produced by co-cultivating explants with *Agrobacterium* tumefaciens harboring a cytidine deaminase fused to nSpCas9 in the T-DNA. Regenerants were selected on culture medium supplemented with IMZ. Analysis of transgene-free plants indicated that transient expression of the T-DNA genes was adequate to induce *ALS* mutations, resulting in IMZ-resistant shoots at a frequency of 11.7%. This research marks the first documentation of T-DNA-free edited citrus plants. While further optimization is necessary to improve editing efficiency, this methodology, combined with *in-planta* transformation techniques, provides a valuable strategy for developing new citrus varieties with enhanced agronomic and organoleptic traits without introducing foreign genetic material.

### 7.3 Cotton

Cotton is one of the most significant cash crops cultivated globally, and its production is often challenged by various biotic and abiotic stresses. Genetic transformation has been instrumental in developing cotton varieties resistant to these challenges (Hussain and Mahmood, 2020). Additionally, genome editing technologies, such as CRISPR/Cas9, enhance these efforts by enabling precise modifications to the cotton genome, allowing for improved resilience and adaptability to environmental stresses while maintaining or enhancing desirable traits.

In this context, Chen et al. (2017) reported the first successful application of CRISPR/Cas9-mediated targeted mutagenesis in cotton, achieved through *Agrobacterium* transformation targeting the shoot apexes. This *in-planta* method allows the screening of transgenic events within 3–4 weeks after genetic transformation. Naked shoot apexes from cotton seedlings were injured with a scalpel. A small cotton ball doused with *Agrobacterium* suspension containing the CRISPR/Cas9 vector was placed in the injured apex. They were then vacuum-infiltrated and co-cultivated. Finally, plants were transferred to a greenhouse, and transgenic events were selected based on antibiotic resistance. This study reported successful editing of the genes *Cloroplastos alterados 1* (GhCLA1) and vacuolar H + -pyrophosphatase (*GhVP*) with efficiencies of 47.6% and 81.8% respectively (Chen et al., 2017). It further demonstrates the feasibility of the CRISPR/Cas9 system for targeted mutagenesis in cotton and its potential to advance functional genomics research by improving molecular cotton breeding.

More recently, Shakoor et al. (2023) reported using an *in-planta* transformation method to obtain stable transformant cotton plants containing the CRISPR/Cas9 genome editing system. The study aimed to develop cotton plants resistant to the cotton leaf curl virus (*CLCuV*), transmitted by the significant crop pest whitefly (*Bemisia tabaci*). Shoot apexes from cotton seedlings were used as explants for transformation, co-cultivated with Agrobacterium tumefaciens for 10 min, and then maintained in a semi-solid culture medium containing antibiotics to prevent contamination for 2–3 days. The plants were transferred to a growth medium for 4–6 weeks. The presence of the CRISPR/Cas9 plasmid was determined by PCR amplification of the backbone vector and Cas9 protein expression was determined and quantified using ELISA assays. This study’s multiplex CRISPR/Cas9 construct contained four sgRNA targeting the plant virus CLCuV: *AC1*, *AC2*, *AC3,* and *beta satellite* (*βC1*) genes. The study reports a transformation efficiency of 1.6% (35/2,150 embryos transformed). The transformed plants exhibited different Cas9 expression levels, from 566.82 ng/mL to 1,305.85 ng/mL. The genetically engineered cotton plants and control plants were infected with the CLCuV, and after 3 weeks, the control plants began to show severe symptoms, in contrast to no symptoms in the transformed plants (Shakoor et al., 2023). Next-generation sequencing was used to determine the knock-out percentages of the proteins targeted by the CRISPR/Cas9 system. The targets associated with the sgRNA1 and sgRNA2 against the *βC1* gene, showed 100% protein variation, including InDels and substitutions. On the other hand, the targets related to the sgRNA3 and sgRNA4 against the DNA-A genes *AC1*, *AC2,* and *AC3* showed more than 90% protein variation, including InDels, substitutions, and frameshifts. Overall, the mutated protein structures are highly dissimilar to wild-type structures (Shakoor et al., 2023). This study shows how CRISPR/Cas9 GE technology, in combination with *in-planta* transformation, can be applied to develop varieties resistant to major crop pests.

### 7.4 Melon

Sasaki et al. (2024) developed a DNA-free, non-culture genome editing method for melon using *in-planta* particle bombardment (iPB-RNP), targeting the CmACO1 and CmGAD1 genes. This innovative technique bypasses the need for tissue culture, overcoming common challenges in genome editing such as genotype dependency and somatic variations. The method yielded a 1.27% and 1.32% mutation rate in the *CmACO1* gene and 1.31% for the *CmGAD1* gene. One of the CmACO1 mutants exhibited an extended shelf life due to reduced ethylene production during fruit ripening, with no noticeable morphological defects. The iPB-RNP method’s effectiveness was confirmed through CAPS analysis and DNA sequencing, showing successful mutations. Ethylene production in the mutant fruits was significantly lower than in the wild-type, extending post-harvest longevity. The technique demonstrated efficiency comparable to genome editing in wheat and barley, making it a promising tool for commercial melon breeding and application across various Cucurbitaceae species.

### 7.5 Peanut

Peanut (*Arachis hypogaea* L.), commonly known as groundnut, is a significant legume crop valued for its seeds, which are rich in edible oil, protein, and fiber (Akram et al., 2018). Advances in tissue culture have incorporated CRISPR/Cas9 (Yuan et al., 2019; Shu et al., 2020; Biswas et al., 2022b; Neelakandan et al., 2022a; Tang et al., 2022), base editing (Neelakandan et al., 2022b), and prime editing (Biswas et al., 2022a) techniques for genetic modifications.

In the context of tissue-independent CRISPR/Cas9 applications, Han et al. (2023) introduced a simple and efficient in-planta transformation method for delivering CRISPR/Cas9 components in peanuts (*Arachis hypogaea* L.), functioning independently of the genotype. The protocol briefly consists of injecting an *Agrobacterium* solution with an OD_600nm_ of 0.6–0.8, containing 100 µM acetosyringone, 10 mM MES, and 10 mM MgCl_2_, in nodes of an adult peanut plant. The CRISPR/Cas9 vector contained an RNA guide targeting the gene *FAD2B* related to the oleic acid content of the peanut seeds. After transformation, the plant was allowed to produce buds and seeds. The seeds were selected according to the oleic acid profile, two seeds showed around 27% higher oleic acid content and were analyzed for putative mutation in the *FAD2B* gene. Mutation in *FAD2B* was detected close to the target site. The stable transformation was confirmed by amplifying the *bar* gene, which is included in the vector. Even if the mutation event might be considered questionable, these results confirmed the efficient delivery of the CRISPR/Cas9 system into the peanut genome by *Agrobacterium* node injection (Han et al., 2023).

Although the protocols for base editing (Yuan et al., 2019; Neelakandan et al., 2022b) and prime editing (Biswas et al., 2022a) do not strictly conform to the definition of in-planta transformation, they can be adapted for such applications, offering an attractive approach for enhancing genetic modifications in crops. Base editing has effectively induced specific point mutations in target genes, such as the *AhFAD2* genes in peanuts, leading to increased oleic acid content. In contrast, prime editing has demonstrated a broader range of editing capabilities, allowing for precise modifications in multiple target genes, including those related to disease resistance and yield traits. Together, these techniques provide promising tools for accelerating crop improvement and developing new varieties with desired characteristics.

### 7.6 Rice

The first *in-planta* transformation method for rice was described by Supartana et al. (2005), utilizing *Agrobacterium*-mediated transformation targeting meristematic tissue from mature embryos. In this method, water-imbibed mature embryos were dipped in an *Agrobacterium* solution, pierced with a needle, and placed on sterile filter paper over wet vermiculite for 9 days, resulting in a stable transformation efficiency of 40% in T_1 plants. Following this foundational work, other research groups developed similar protocols with modifications, such as immersing tissue in bacterial solution (Hanjagi et al., 2011), employing vacuum infiltration (Lin et al., 2009), incorporating acetosyringone to enhance *Agrobacterium* infection (Naseri, 2012), and using surfactants like Tween 20 to improve bacterial penetration (Ahmed et al., 2018). These methods reported varying transformation efficiencies ranging from 8% to 40%.

In a more recent study, Karthika et al. (2021) employed an *in-planta Agrobacterium* transformation method to deliver CRISPR/Cas9 components targeting the indica rice DNA mismatch repair gene *MSH2*. Embryonic shoot apical meristems from pre-germinated seedlings were pierced with a needle and incubated with an *Agrobacterium* culture supplemented with acetosyringone (150 µM) for 1 hour. The surviving seedlings (16.2%) were putatively selected as transformed plants at T0, and PCR confirmed successful transgene integration in 27 plants. The disruption of the *MSH2* gene, which theoretically reduces DNA mismatch repair events and facilitates the creation of insertions and deletions (InDels), was confirmed by sequencing in one of seven plant lines at T2. The resulting mutant line exhibited significantly longer panicles, more spikelets per panicle, and more seeds per spikelet than the wild type, suggesting the potential for these mutant indica rice lines to act as donor lines for stabilizing desirable traits in breeding.

In exploring new delivery methods, Demirer et al. (2021) have shown the use of nanoparticles loaded with exogenous DNA for plant cell transformation. Dunbar et al. (2022) further developed a rice *in-planta* transformation method using carbon nanotubes (CNTs) loaded with various DNA reporter vectors and a CRISPR/Cas9 vector. They used carboxylic acid-functionalized CNTs modified with polyethyleneimine (PEI) to facilitate the attachment of plasmid DNA. The CNTs were infiltrated into mature rice leaves through mechanical wounding and soaking in a pDNA-PEI-CNT solution. After incubating rice embryos from mature seeds to initiate germination, shoot tips were cut to expose the shoot apical meristems (SAMs) and subsequently vacuum-infiltrated in the CNT solution. Six vector constructs of varying sizes were tested using reporter genes *GFP*, *YFP*, and *GUS*, showing efficient expression in plant cells confirmed by transcript analysis. The CRISPR/Cas9 vector targeting the *OsPDS* gene was attached to the CNTs and delivered to rice seeds and isolated embryos. Out of 1,120 seeds and 112 embryos treated, phenotypic alterations such as partially albino leaves and stunted growth were observed in 121 (10.8%) seeds and 13 (11.6%) embryos. Although sequencing detected several putative InDels in 33 plants, the authors noted high chimerism and low mutation frequency, suggesting further optimization is necessary for this nanotechnology-based CRISPR/Cas9 delivery system to overcome the bottlenecks of plant tissue culture.

Lastly, recent research has combined in-planta transformation methods for rice with modern genetic engineering technologies, including microRNA (Faisal et al., 2017) and interference RNA (Wahyuningtyas et al., 2016), showcasing the evolving landscape of genetic modifications in rice.

### 7.7 Soybean

The clustered regularly interspaced short palindromic repeat (CRISPR) systems, particularly those involving the Cas9 and Cas12a proteins, remain the most widely used technologies for plant genome editing due to their ease of use and versatility (Pickar-Oliver and Gersbach, 2019). To further expand the functionality of CRISPR-based systems, novel strategies such as CRISPR-associated protein engineering have been explored.

Nagy et al. (2022) developed a CRISPR-based approach to enhance the efficiency of site-directed DNA integration in plants by fusing the LbCas12a endonuclease with the HUH endonuclease from the Faba Bean Necrotic Yellow Virus (FBNYV). This method, initially reported in human cell lines (Aird et al., 2018), was adapted for soybeans to increase the concentration of donor DNA at the site of double-strand breaks (DSBs). The preassembled RNP/ssDNA complex used for in-planta transformation involved purified fusion proteins (LbCas12a:linker or HUH:linker), CRISPR RNA targeting the D5 region of the soybean genome, and a 70-nt ssDNA oligonucleotide donor template homologous to the D5 region with or without the FBNYV origin sequence. Various treatments were tested to evaluate the system’s efficiency, and following the selection of the best combination, particle bombardment was used to deliver the genome editing components into soybean embryos. Of the 594 T0 transformants generated, 70% exhibited targeted insertions or deletions, and the integration rate of the donor DNA reached 25.9%, which was four times higher than the control. This demonstrated the potential of fusion proteins between CRISPR-associated proteins and viral endonucleases to enhance site-directed integration in plants using in-planta methods.

Meanwhile, Viswan et al. (2022) developed a direct delivery system for CRISPR/Cas9 RNP complexes in soybeans using an automated microneedle array (MNA). This DNA-free *in-planta* transformation method provides a scalable and high-throughput approach for genome editing. The MNA system was first tested on *A. thaliana* leaves to evaluate delivery efficiency, where Cre recombinase was used as a reporter. The successful delivery was confirmed by GUS staining, with all leaves showing positive results for Cre recombination. The same system was then used to deliver CRISPR/Cas9 components to soybean shoot apical meristems (SAMs), targeting the gene for carotenoid biosynthesis (*PDS11/18*). Although the mutation rate was low (0.03%), the authors estimated that the frequency of deletions in meristem cells where the needle was inserted was around 6%.

Kuwabara et al. (2024) introduced another genome editing technique for soybeans, employing the *in-planta* bombardment-ribonucleoprotein (iPB-RNP) method. This technique eliminates the need for foreign DNA or traditional tissue culture processes, such as embryogenesis or organogenesis. The shoot apical meristem (SAM) of soybean embryonic axes was targeted because of its stem cells, which later develop into germ cells during the reproductive phase. By delivering the CRISPR/Cas9 RNP complex into SAM stem cells via particle bombardment, the researchers successfully generated genome-edited plants. The technique targeted the allergenic Gly m Bd 30K gene, with mutation rates between 0.4% and 4.6% in different soybean varieties. The mutations were inherited by the E1 generation, and simultaneous mutagenesis at other loci was also achieved. This iPB-RNP method offers a practical, DNA-free solution for precise genome editing in soybean and other dicotyledonous plants, bypassing the need for labor-intensive tissue culture techniques.

### 7.8 Wheat

Wheat is considered one of the most challenging major cereal crops to transform. Despite its importance as a global staple, genetic engineering applications in wheat lag behind those in rice and maize (Hamada et al., 2017). As a result, efforts have been made to develop alternative transformation techniques, particularly for elite commercial cultivars that have proven resistant to tissue culture-based methods.

Hamada et al. (2017) introduced an in planta biolistic transformation technique using shoot apical meristems (SAMs) from mature embryos as target cells, delivering gene expression constructs via gold particles. Bombardments were performed four times using helium pressure of 1,100 or 1,350 psi from a distance of 6 cm. This method successfully achieved transient expression, stable integration, and F1 inheritance in the model wheat cultivar “Fielder” and the elite Japanese cultivar “Haruyokoi.” Later, the method was adapted for genome editing by delivering CRISPR/Cas9 constructs (Hamada et al., 2018). Targeting the TaGASR7 gene, involved in grain weight and length regulation, led to mutations in 5.2% of T₀ plants, with some plants showing heterozygous mutations in the hexaploid wheat’s A, B, and D genomes. In the T₁ generation, three plants were identified as homozygous mutants across all genomes. A DNA-free variant of this method, using CRISPR/Cas9 RNP complexes, also showed editing efficiency at around 3% (Imai et al., 2020).

This in planta biolistic method was further applied to edit commercially important Japanese wheat cultivars “Haruyokoi,” “Yumechikara,” and “Kitanokaori” (Liu et al., 2021). Targeting the TaQsd1 gene, which is linked to seed dormancy and pre-harvest sprouting, resulted in mutations in 2.5% of bombarded embryos. Chimerism required the analysis of all T₁ plants, and one plant was identified as heterozygous across the three genomes, with a homozygous T₂ individual displaying a delayed seed germination phenotype. This method allowed for the rapid improvement of elite wheat cultivars using CRISPR/Cas9.

Building on Hamada et al.’s method, researchers recently applied *in-planta* particle bombardment to deliver CRISPR/Cas9 RNPs into wheat SAMs. The transformants were screened using cleaved amplified polymorphic sequence (CAPS) assays, with an editing efficiency of 8.3% in T₀ plants, including a plant with mutations across all three homologous genes. This efficiency is comparable to DNA-based delivery (Kumagai et al., 2022).

In the same study, Kumagai et al. (2022) targeted the wheat orthologs of TaSD-A1, TaSD-B1, and TaSD-D1, genes homologous to rice’s sd1 gene, which encodes GA20 oxidase and is involved in gibberellin synthesis. Using a cultivar carrying the Rht1 semidwarf allele, researchers aimed to develop a wheat line carrying both Rht1 and tasd1 mutations. The CAPS assay identified 6.9% of bombarded embryos as positive for editing, and T₁ sequencing revealed mutations in all three genomes in a plant named H7-1 E1. These mutations silenced the TaSD1 genes, producing mutant plants with a 10% reduction in height and greener leaf color compared to wild-type.

The *in-planta* particle bombardment (iPB) for wheat SAMs transformation and genome editing was first reported by Hamada et al. (2017). This strategy has been used repeatedly by this group of collaborators (Hamada et al., 2018; Imai et al., 2020; Liu et al., 2021; Kumagai et al., 2022), successfully improving the efficiency of the system and expanding its application. More recently Luo et al. (2024) describe this strategy in a protocol article, currently this method results in 3%–5% of T₀ plants carrying mutant alleles, with 1%–2% successfully passing these alleles to the next-generation. Their work showcases the potential of bypassing the need of tissue culture for genome editing in wheat varieties that are difficult to transform.

## 8 Novel genome editing methods that bypass tissue culture steps

### 8.1 *In-planta* CRISPR/Cas9 genome editing through *de novo* meristem induction

Maher et al. (2020) introduced an innovative strategy to overcome the tissue culture barrier in genome editing by using ectopic expression of developmental regulators (DRs) to induce meristem formation in somatic cells. By co-expressing DRs like WUSCHEL2 (*WUS2*) and SHOOT MERISTEMLESS (*STM*) alongside CRISPR/Cas9 components, the researchers were able to reprogram genome-edited somatic cells into meristems, which could then be regenerated into full plants. The initial experiments were performed in *N. benthamiana*. A key development in this research was the Fast-TrACC (fast-treated Agrobacterium coculture) method, a rapid and highly efficient *Agrobacterium*-mediated transformation technique for transient expression. Researchers tested 12 combinations of DR-encoding constructs with a luciferase reporter, and the highest rate of meristem-like structures (30%) was achieved using a combination of *WUS2* and *STM*. Genome editing was successfully performed by incorporating sgRNA targeting the Phytoene desaturase (*PDS*) gene into Cas9-expressing transgenic plants, with the edited plants regenerated under aseptic conditions. In a second approach, DR and sgRNA constructs were injected into wounds of soil-grown Cas9-expressing plants where natural meristems had been removed, inducing new shoots with the expected photo-bleaching phenotype from PDS gene disruption (15%). This technique was further applied successfully to other species, including tomato, potato, and grape, using the Fast-TrACC method to generate *de novo* transgenic shoots. The potential of this method to induce meristems via different delivery techniques opens the door for expanding *in-planta* transformation and genome editing to a wider range of plant species, bypassing the traditional tissue culture bottleneck.

### 8.2 Spray-on CRISPR genome editing

Doyle et al. (2019) introduced a straightforward method for spray-on genome editing, which has the potential to become a simple, fast, cost-effective, and potentially universal approach for plant genetic modification. This technique uses carbon dots coated with a plasmid containing CRISPR/Cas9 components, which are then sprayed onto plant leaves. The plasmid encodes a *GFP* reporter gene with a nuclear localization sequence, along with the Cas9 gene and a sgRNA targeting the wheat *SPO11* gene. Fluorescent-marked tissue confirmed transient transformation following the foliar spray, with a transformation efficiency of 27.7% in wheat. Additionally, transient expression was observed in maize, barley, and sorghum. A 250 bp deletion in the wheat *SPO11* gene was confirmed by PCR and DNA sequencing. This spray-on method highlights the versatility of carbon dot-DNA complexes in delivering genome-editing components to mature plant cells for transient transformation. The use of nanoparticles for CRISPR/Cas9 delivery is an emerging area of research (Alghuthaymi et al., 2021) with significant potential for advancing plant biotechnology and improving commercially important crops.

### 8.3 Regenerative activity-dependent *in-planta* injection delivery

Mei et al. (2024) developed the regenerative activity-dependent *in-planta* injection delivery (RAPID) method to address the challenges of low efficiency and complex protocols associated with traditional plant genetic transformation techniques. RAPID takes advantage of a plant’s natural regenerative capacity by delivering *A. tumefaciens* directly into the meristems of plants, which then induces transformation in nascent tissues. Stable transgenic plants are subsequently obtained through vegetative propagation of the transformed tissues, bypassing the need for tissue culture procedures. This method was validated by editing the Phytoene desaturase (*PDS*) homolog in potato (*Solanum tuberosum*), demonstrating its effectiveness for genome editing in plants. RAPID also showed success in transforming multiple genotypes of sweet potato (*Ipomoea batatas*) and bay hops (*Ipomoea pes-caprae*), all species with strong regeneration capacities. Compared to conventional transformation methods, RAPID achieved significantly higher transformation efficiency and a shorter process duration, making it a valuable alternative for plant species capable of active regeneration.

The RAPID method is crucial because it streamlines *in-planta* transformation and genome editing, especially for economically important crops like potatoes and sweet potatoes. By eliminating the dependency on labor-intensive tissue culture steps, RAPID can facilitate more rapid genetic modification, making it an up-and-coming tool for advancing molecular breeding and genetic engineering in various crops. This opens the door to improving essential traits like yield, pest resistance, and climate resilience in key agricultural species.

### 8.4 Cut-dip-budding delivery system

Cao et al. (2022) introduced an innovative Cut-Dip-Budding (CDB) delivery system to address the limitations of current gene delivery systems, which restrict genetic modification to fewer than 0.1% of plant species. Traditional methods often require time-consuming and costly tissue culture processes, even for species that can be transformed. In contrast, the CDB method uses *A. rhizogenes* to inoculate plant explants, generating transformed roots. These roots, through suckering, produce transformed buds, enabling heritable transformation. The CDB system was successfully applied to a diverse range of plant species across multiple families, including herbaceous plants like *Taraxacum kok-saghyz* and *Coronilla varia*, the tuberous root plant sweet potato (*Ipomoea batatas*), and woody plants such as *Ailanthus altissima*, *Aralia elata*, and *Clerodendrum chinense*. These species, previously difficult or impossible to genetically modify, were efficiently transformed or gene-edited using the CDB method. Notably, the procedure was carried out under non-sterile conditions and did not require tissue culture, significantly simplifying the process.

The CDB system’s success in overcoming transformation barriers suggests its potential for broad application across various plant species. This would make large-scale genetic modification more accessible and cost-effective. This method could be crucial in enhancing crop improvement and plant biotechnology, particularly in species previously considered recalcitrant to transformation.

In this context, Tamizi et al. (2023) explored an *in-planta* transformation protocol for genome editing in plants using the CRISPR/Cas9 system, focusing on two standard methods: *Agrobacterium*-mediated transformation and particle bombardment. Both approaches typically involve intricate manipulations of undifferentiated cells and tissue culture to regenerate edited plants, making the process time-consuming and labor-intensive. To address this, the researchers developed a simplified, tissue culture-independent protocol for delivering CRISPR/Cas9 directly through *in-planta* transformation in Malaysian rice (*Oryza sativa* L. subsp. indica cv. MR 219). They achieved a 9% transformation efficiency by targeting sprouting seeds with cut coleoptiles for infiltration by *Agrobacterium tumefaciens*. In the procedure, dehusked seeds were surface-sterilized, imbibed, and their coleoptiles cut to expose the apical meristem, which was then inoculated with *A. tumefaciens* strain EHA105 carrying the CRISPR/Cas9 expression vector. After five to 6 days of co-cultivation in a dark room at 25°C ± 2°C, the plants were subjected to rooting, acclimatization, and growth phases. Two months later, leaves were screened for hygromycin resistance, and resistant plants were considered potential transformants. Polymerase chain reaction (PCR) confirmed the integration of the Cas9 gene in four T0 plants, and further analysis during the fruiting stage verified the gene’s presence in three randomly selected tillers from two transformed plants. This protocol offers a rapid, tissue culture-free method for editing the rice genome, marking rice’s first CRISPR/Cas9 in-planta transformation report.

## 9 Challenges of *in-planta* CRISPR/Cas9 genome editing

Recent reviews of CRISPR/Cas9 plant genome editing highlight its technological advancements, applications, challenges, and future possibilities in agriculture (Cardi et al., 2023). However, several constraints remain, including the lack of efficient delivery methods, species- and genotype-dependent transformation barriers, identification of promising gene targets, improving editing and multiplexing efficiencies, minimizing off-target effects, developing standardized regulatory procedures for testing and approval, and enhancing public acceptance of this technology (Yang, 2020; Anjanappa and Gruissem, 2021; Zhang et al., 2021; Kumar et al., 2021; Le et al., 2021).

Delivering CRISPR/Cas9 reagents and plant regeneration remains a significant hurdle among these challenges. Efficient delivery methods that bypass tissue culture steps are frequently mentioned as crucial for advancing genome editing. While *in-planta* transformation methods offer potential, they also have limitations. The floral dip method, for example, has a low transformation rate, particularly in species with low seed production. Additionally, some *in-planta* protocols suffer from low reproducibility, highlighting the need for further optimization and standardization in transformation efficiency. CRISPR/Cas9 edits occur at the single-cell level, often leading to chimerism when embryos or meristematic tissue are transformed. One strategy to address this is the co-selection of CRISPR-induced mutations in both the target and marker genes. For instance, Rinne et al. (2021) utilized a multiplexing CRISPR/Cas9 system targeting the *MAR1* marker gene, which confers kanamycin resistance. This co-selection improved the efficiency of mutation screening in a second target gene (Rinne et al., 2021). Another potential approach involves high-throughput automated phenotyping using imaging (Sorrentino et al., 2021), which could be helpful when the target gene edit results in an altered phenotype.

## 10 Conclusion and future prospects

Plant transformation remains essential in biotechnology and modern breeding, particularly for CRISPR/Cas9 genome editing. Achieving efficient delivery of CRISPR/Cas9 components while bypassing tissue culture would represent a breakthrough, allowing greater focus on other challenges, such as minimizing off-target effects, enhancing multiplexing, improving editing efficiency, and fine-tuning gene expression.

*In-planta* transformation methods have been developed as alternatives for species or genotypes resistant to traditional tissue culture approaches. However, further research is needed to extend these methods beyond common model plants like *Arabidopsis thaliana* and *Nicotiana benthamiana*, which are usually transformed via floral dip. This review highlighted the potential of this strategy for commercially valuable crops like camelina, citrus, cotton, peanut, rice, soybean, and wheat.

*In-planta* transformation also expands the possibilities for genome editing and biotechnology across a broader range of species. By simplifying the application of CRISPR/Cas9 systems, this approach could make the technology more accessible to researchers, particularly in developing countries. It could enable local solutions to agricultural challenges, unlocking new opportunities. Advancing *in-planta* transformation and CRISPR/Cas9 genome editing offers tremendous potential for achieving the United Nations Sustainable Development Goals (SDGs), particularly in enhancing food security, promoting sustainable agriculture, and fostering climate-resilient crops. These technologies can expedite the development of crops with traits like drought tolerance, pest resistance, and improved nutritional quality, which are critical for adapting agriculture to climate change. Creating climate-resilient crops will help mitigate extreme weather impacts and promote more sustainable farming systems, reducing reliance on chemical inputs and enhancing biodiversity. As these methods become more accessible, they can empower communities and researchers globally to tackle region-specific challenges, contributing to a more sustainable and resilient agricultural future.
